# Obesity and Bone Mineral Density Protection Paradox in Chronic Kidney Disease: Secreted Protein Acidic and Rich in Cysteine as a Piece of the Puzzle?

**DOI:** 10.3390/life13112172

**Published:** 2023-11-06

**Authors:** Abdelaziz Ghanemi, Fabrice Mac-Way

**Affiliations:** 1 Endocrinology and Nephrology Axis, L’Hôtel-Dieu de Québec Hospital, CHU de Québec Research Center, Quebec, QC G1R 2J6, Canada; abdelaziz.ghanemi.1@ulaval.ca; 2 Department of Medicine, Faculty of Medicine, Laval University, Quebec, QC G1V 0A6, Canada

**Keywords:** secreted protein acidic and rich in cysteine (SPARC/osteonectin), obesity paradox, chronic kidney disease, bone density protection

## Abstract

Obesity is a health condition that represents a risk factor for numerous diseases and complications. However, obesity might also have—to some extent—some “benefits” in certain situations. This includes potential bone protection in patients suffering from chronic kidney disease. In an attempt to explain such a paradox, we highlight secreted protein acidic and rich in cysteine (SPARC) as a hypothetical mediator of this protection. Indeed, SPARC properties provide a logical rationale to describe such bone protection via its overexpression combined with its calcium-binding and collagen-binding properties. We believe that exploring such hypotheses could open new doors to elucidate unknown pathways towards developing a new generation of molecular therapies.

One of the biggest challenges limiting our understanding of the diseases, and therefore, the development of therapeutic options is the unknown mechanisms and the poor understanding of the related underlying pathways. In addition, the existence of biological and medical paradoxes further complexifies such a challenge. Biological pathways, biochemical reactions, and medical observations follow patterns and features that represent the bases established through empirical observations or epidemiological conclusions on which biomedical theories are built. This allows us to predict a pathological outcome, a drug effect, or a disease prognosis. However, there are concepts that defy these biomedical patterns and features. Such observations are referred to as paradoxes. An example worth exploring is obesity paradoxes in chronic kidney disease (CKD), also named reverse epidemiology [1], that usually indicate the benefits of obesity on survival [2,3], indicating an all-cause mortality (including cardiovascular disease) reduction in CKD patients suffering from obesity compared to those with normal body weight/fat mass.

Obesity represents a serious health problem with heavy consequences at both societal and economic levels. Obesity development, which has been compared to cancer [4], is correlated with increased risk for a variety of diseases and health problems such as cardiovascular diseases [5], type 2 diabetes [6], cancer [7], metabolic disorders [8], nonalcoholic fatty liver disease [9], obstructive sleep apnea [10], coronavirus disease-2019 (COVID-19) [11], and CKD [12,13]. Paradoxically, numerous studies have also indicated protective or beneficial impacts that obesity would have in the context of various pathologies, health problems, and even ageing [14,15,16,17,18,19]. Within this short piece of writing, we specifically aim to shed light on the paradox described in the context of obesity and bone protection in CKD.

Bone disorders can be the consequence of either physiological changes, including ageing [20,21], or pathological statuses/diseases such as CKD [22,23,24,25,26,27]. As a consequence of bone diseases, bone mineral density can be affected, leading to an increased risk of bone fracture [28]. Obesity is frequently associated with higher bone mineral density [29] and is, historically, believed to protect against osteoporosis [30]. On the other hand, obesity has also been associated with higher fracture risk [31,32].

Herein, we provide the rationale behind suggesting secreted proteins that are acidic and rich in cysteine (SPARC/osteonectin/BM40) as part of the mechanistic links between obesity and possible bone protection in diseases that are supposed to negatively impact bone homeostasis, such as CKD. Among the non-collagenous proteins, SPARC is the most expressed in mineralized tissues [33]. It is also expressed in various tissues and plays roles in diverse biological functions and processes [34].

SPARC expression increases in various situations, such as obesity [35], where it is produced by the adipose tissue [36,37], and in newly diagnosed type 2 diabetes mellitus patients, there is a correlation between both the body mass index (BMI) and fat percentage and SPARC plasma levels [38]. In addition, with obesity tending to increase muscle mass [39], the SPARC increase in obese subject sera could also be a result of increased muscle mass since SPARC is a myokine as well [40,41]. Therefore, we hypothesize that obesity-induced SPARC overexpression in both tissues and serum could explain—at least in part—such bone protection in the context of CKD and other diseases as compared to non-obese patients suffering from the same conditions.

This hypothesis is based on the calcium-binding and collagen-binding properties of SPARC [42,43] that would strengthen bone structure. In fact, SPARC (also known as osteonectin) has been described as a bone-specific biomolecule that links collagen to minerals (mineralized collagen) [43]. Knowing the roles of both calcium [44] and collagen [45] in bone structure and strength, SPARC would enhance such cohesion and improve bone density. The role of SPARC in strengthening bones would be based on the affinity of this glycoprotein to bind both collagen [46] and calcium [47]. This will increase the building up of the various biological components of the bones, especially knowing the importance of SPARC within both the mineralized tissue [33] and the extracellular matrix [48,49,50] that is responsible for cellular adhesion and tissue connections.

Furthermore, SPARC-deficient mice both develop osteopenia and have decreased bone formation and osteoblast number [51,52,53], confirming the important role SPARC has in bone formation [33]. This hypothesis is also in accordance with the fact that SPARC has also been characterized as an exercise-induced gene [54,55], since the exercise-induced benefits on bone [56,57] could also be (at least in part) mediated with SPARC.

However, to explain why the other properties of SPARC (such as the metabolic effects [58], cancer inhibitor [40,59,60], regenerative factor [61], and anti-inflammatory [62]) are not increased with SPARC overexpression, we have previously suggested that for such properties, a resistance develops as it would require putative receptors and intracellular pathway activation. On the other hand, the effect SPARC would have on bone would be further maintained and not be affected by such resistance because SPARC interactions would rather be chemical through its calcium-binding and collagen-binding properties. The fact that SPARC has a calcium-binding site and collagen-binding properties would allow it to strengthen extracellular matrix calcification and, therefore, increase bone mineralization and also the vascular calcification that is very prevalent in CKD patients [63]. This points out the possible negative effects of SPARC on the development of vascular calcification that also result from the same properties (calcium-binding site and collagen-binding) that lead to bone protection. How SPARC may be differently involved in the process of vascular calcification between the obese and non-obese populations in CKD remains worth exploring.

The hypothesis we have previously provided [58] about the importance of SPARC overexpression is that it would be a balancing mechanism. Indeed, following obesity development and the dysregulation of energy homeostasis, SPARC overexpression would be a mechanism aiming to restore metabolic balance and reverse obesity. This would be mediated with the properties that SPARC has in terms of metabolic enhancement within the various key metabolic tissues (mainly adipose tissue and muscles). However, such “corrective” metabolic pathways would be inefficient as “SPARC resistance” would develop with obesity establishment in a way similar to insulin resistance [64] seen during diabetes. This indicates the evolutionary significance of providing a metabolic adaptive advantage aiming to protect energy homeostasis. Such significance is supported by the fact that SPARC, which is an evolutionarily conserved glycoprotein [46], is also highly conserved between vertebrates and invertebrates [47].

What further supports the hypothesis that SPARC is related to bone protection is the fact that vascular calcification can develop in CKD patients regardless of their obese or non-obese status, but the vascular calcification is more pronounced in obese patients with lower kidney function [65]. This points to obesity-induced SPARC overexpression as a potential explanation. Such a phenotype of vascular calcification reflects strong mineralization through calcification that would be mediated with SPARC mainly via its calcium-binding properties. Indeed, in vitro and ex vivo studies show that SPARC is expressed during vascular calcification [66], which fits with our presented hypothesis. It is worth mentioning that the obesity paradox has also been observed in osteoporosis [31], liver cirrhosis, heart failure, elderly individuals, chronic obstructive lung disease, and metastatic cancer [67]. This phenomenon might also be explained in part by the properties of SPARC towards improving general homeostasis, which highlight the importance of further exploring this multifunctional protein.

Figure 1 summarizes the key concepts related to the hypothesis we present herein. We believe these ideas will trigger further exploration of SPARC in the context of obesity, bone diseases, and CKD for a better understanding of the different paradoxical phenotypes, allowing for the development of potential novel therapeutic approaches. This requires a multidisciplinary approach involving the exploration of bone marrow adipogenesis, the type of obesity (visceral abdominal fat versus subcutaneous fat), and the low-grade systemic inflammation that characterizes obesity. The variabilities in SPARC implications in inflammation in/and the context of obesity [68,69,70] remain an important piece to explore and add to this paradoxical puzzle.

The paradox and the variations in data suggesting bone protection and others indicating bone fragility with obesity could be explained by the type of obesity. Indeed, beyond both the body weight and the BMI, the obesity phenotype and the adiposity distribution are extremely important determinants of the pathological outcomes and the obesity consequences on the homeostasis of different tissues, including bones and kidneys. For instance, intrarenal fat accumulation (adiposity distribution) has been suggested to cause obesity-related CKD [71], and subcutaneous fat might have different effects on bones compared to visceral abdominal fat [30].

Furthermore, such obesity-dependent bone protection could also be site-dependent [30] rather than systemic. Therefore, such interaction would mainly depend on both the obesity phenotype and bone location. The importance of highlighting such an obesity paradox derives from the fact that obesity represents the epidemic of our era, which is expected to continue increasing [71]. Finally, such mechanistic links can represent starting points towards developing therapies based on SPARC or on the molecular targeting of SPARC-related pathways, as we have already suggested in diverse pathological contexts [72,73,74]. However, the gap is still large, as further experiments are required to investigate the hypothesis of obesity and the bone mineral density protection paradox involving SPARC as the link among other biomolecules. For instance, osteopontin could also be molecularly involved in the link explaining the obesity paradox in CKD. Similarly to SPARC, osteopontin levels increase with obesity [75] and decrease with exercise-induced fat loss [76]. It has important roles in bone homeostasis and metabolism [77]. Osteopontin contributes to the differentiation, proliferation, and adhesion of various bone-related cells [78], calcium and phosphate metabolism regulation [79], and has roles in bone mineralization [79]. For vascular calcification, however, whereas an acute increase in osteopontin ameliorates vascular calcification, a chronic increase in osteopontin is related to negative cardiovascular outcomes [80]. In addition, osteopontin structure is different from SPARC as it is highly phosphorylated and rich in aspartic acid [81]. Thus, it suggests a mechanistic pathway different from those of SPARC, as it, like SPARC, contributes to bone protection but has different potential effects with regard to vascular calcification. Such differences in impacts might be explained by an environment-dependent effect. Indeed, at the beginning of obesity development, the initial increases in osteopontin would be an attempt to “reverse” or reduce the impact of obesity on vascular calcification. However, once obesity is established (chronic increases in osteopontin), a new biological environment is established (inflammation, metabolic disorder, signalling molecules, etc.), shifting the impact of osteopontin towards worsening the cardiovascular phenotype. Importantly, osteopontin’s possible implications in the obesity paradox might not only be due to obesity, as CKD alone can increase circulating levels of osteopontin and kidney expression as well [79]. This suggests that the protective level cannot be reached via CKD-induced overexpression, and the overexpression due to obesity combined with that of CKD provides a sufficient osteopontin levels to induce a protective level, contributing to the obesity paradox in CKD.

Understanding such obesity paradoxes might reveal previously unknown molecular pathways and allow the identification of potential therapeutic targets to improve CKD outcomes for patients, especially those suffering from obesity. Finally, it is worth mentioning that obesity-related outcomes do not only depend on body weight or even fat percentage but also on fat distribution [82,83], as—for instance—visceral adiposity and ectopic adiposity [84] have a worse outcome than subcutaneous adiposity. Therefore, the obesity paradox needs to be “adjusted” depending on the fat distribution.

## Figures and Tables

**Figure 1:**
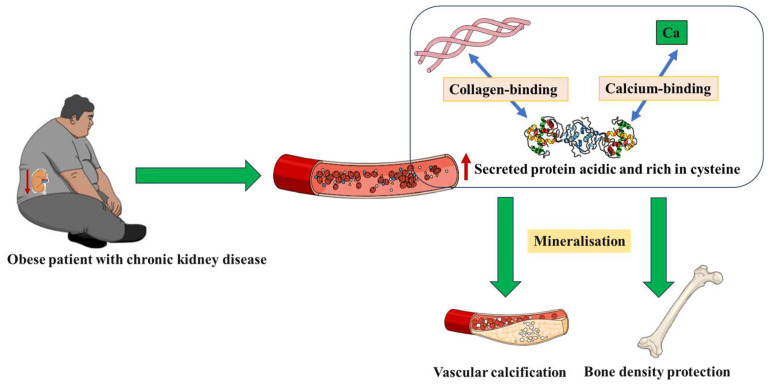
Hypothetical mechanisms of how the obesity-induced increased secreted protein acidic and rich in cysteine (SPARC) expression protects bones from chronic kidney disease-related density loss as well as SPARC contribution to vascular calcification.

## Data Availability

Not applicable.
